# Development of a Comprehensive Decision Support Tool for Chemotherapy-Cycle Prescribing: Initial Usability Study

**DOI:** 10.2196/62749

**Published:** 2025-03-31

**Authors:** Sanna Iivanainen, Reetta Arokoski, Santeri Mentu, Laura Lang, Jussi Ekström, Henri Virtanen, Vesa Kataja, Jussi Pekka Koivunen

**Affiliations:** 1 Department of Oncology and Radiotherapy, Oulu University Hospital, Kajaanintie 50, Oulu, 90220, Finland, 358 83153038; 2 Kaiku Health Oy, Helsinki, Finland

**Keywords:** cancer, chemotherapy, ePRO, electronic patient-reported outcome, decision support system

## Abstract

**Background:**

Chemotherapy cycle prescription is generally carried out through a multistep manual process that is prone to human error. Clinical decision support tools can provide patient-specific assessments that support clinical decisions, improve prescribing practices, and reduce medication errors.

**Objective:**

We hypothesized that a knowledge-based, patient-derived, evidence-directed decision support tool consisting of multiple modules focusing on the core duties preceding chemotherapy-cycle prescription could result in a more cost-effective and error-free approach and streamline the workflow.

**Methods:**

A 1-arm, multicenter, prospective clinical trial (“Follow-up of Cancer Patients Receiving Chemotherapy or Targeted Therapy by Electronic Patient Reported Outcomes-tool” [ECHO] 7/2019-1/2021; NCT04081558) was initiated to investigate the tool. The most important inclusion criteria were the presence of colorectal cancer (CRC) treated with oxaliplatin-based chemotherapy, age ≥18 years, Eastern Cooperative Oncology Group [ECOG] performance score of 0 to 2, and internet access. A decision support tool that included digital symptom monitoring, a laboratory value interface, and treatment schedule integration for semiautomated chemotherapy cycle prescribing was integrated into the care pathway. Performance was assessed by the percentage of chemotherapy cycles with sent and completed symptom questionnaires, while perceptions of health care professionals (HCPs) on the feasibility of the approach were collected through a 1-time semistructured interview.

**Results:**

The ECHO trial included 43 patients with CRC treated with doublet or triplet chemotherapy in an adjuvant or metastatic setting. Altogether, 843 electronic patient-reported outcome (ePRO) symptom questionnaires were completed. Of the 15 recorded symptoms, fatigue (n=446, 52.9%) and peripheral neuropathy (n=429, 50.9%) were reported most often, while 137 grade 3 to 4 symptoms were recorded, of which diarrhea (n=5, 4%) and peripheral neuropathy (n=4, 3%) were the most common. During the study, 339 chemotherapy cycles were prescribed, and for the 77% (n=262) of new chemotherapy cycles, ePRO questionnaire data were available within preset limits (completed within 3 days prior to chemotherapy scheduling) while 65% of the cycles (n=221) had symptom questionnaire grading at ≤1%, and 67% of the cycles (n=228) had laboratory values in a preset range. The recommendations by the tool for a new chemotherapy cycle were tier 1 (green; meaning “go”) in 145 (42.8%) of the cycles, tier 2 (yellow; “evaluate”) in 83 (25%), and tier 3 (red; “hold”) in 111 (32.7%). HCPs (n=3) were interviewed with a questionnaire (comprising 8 questions), revealing that they most valued the improved workflow, faster patient evaluation, and direct messaging option.

**Conclusions:**

In this study, we investigated the feasibility of a decision support system for chemotherapy-cycle pre-evaluation and prescription that was developed for the prospective ECHO trial. The study showed that the functionalities of the investigated tool were feasible and that an automated approach to chemotherapy-cycle prescription was possible for nearly half of the cycles.

## Introduction

In health care, digital approaches can potentially improve accessibility, increase the comprehensiveness of care, and streamline processes in a cost-effective manner. Digital tools are especially convenient in facilitating communication independently of time and place, as well as facilitating duties requiring repeated numerical comparison [1-3]. In oncology, electronic patient-reported outcome (ePRO)–based monitoring has gained a great deal of interest in recent years. ePROs have been shown to improve quality of life (QoL) and survival and reduce the number of unscheduled visits among patients receiving chemotherapy for advanced cancer [4-6]. Reasons for the observed beneficial effects of ePROs are not well known but might be related to urgency algorithms, long-term symptom data, better management of side effects, symptom control, patient empowerment, and facilitated communication between the patient and care team [7-11].

For cytotoxic chemotherapy, the most limiting factors for dose intensity are generally bone marrow function, neuropathy, fatigue, and nausea. Dose intensity typically correlates to treatment outcomes [12], and methods to maintain the intensity level with a personalized approach could improve treatment. For monitoring of dose-intensity limiting factors, both laboratory values and symptom profiles are required.

In oncology units, prescription of a new chemotherapy cycle is based on general laboratory values such as neutrophil counts and transaminases being in the selected reference range and the absence of severe symptoms indicating treatment side effects or cancer progression. In most units, precycle assessment is done by phone-based symptom evaluation and manual comparison of laboratory values to a safety reference. ePRO-collected symptoms can be converted to numerical data, which enables automated comparison to a safety reference scale; the quality of automated comparison surpasses manual review.

Delivering evidence-based, personalized care that involves patients requires profound changes in the traditional, hospital organization–based structure, process, and organization of care, along with clinically validated incentives to support such changes. Health care providers must engage and apply a vast amount of scientific information to provide high-quality patient care. Decision support tools can provide patient-specific assessments that support clinical decisions, improve prescribing practices, and reduce medication errors, and these tools could be part of a solution to the increasing cognitive burden currently placed on clinicians [13].

Clinical decision support systems (CDSSs) have been classified and subdivided into various categories, frequently as knowledge based or non–knowledge based. In knowledge-based systems, outputs are based on rules (IF-THEN statements) relying on literature-based, practice-based, or patient-directed evidence. Similarly, CDSSs that are non–knowledge based require a data source, but these systems use artificial intelligence (AI) methodology in work-up rather than being programmed to follow expert medical knowledge. Non–knowledge-based CDSSs, although a rapidly growing use case for AI in medicine, come with challenges including problems understanding the logic that the AI uses in creating recommendations (the so-called black box issue) and problems with validated data availability [14,15].

Taking into consideration the present hurdles in the use of non–knowledge-based CDSSs in clinical practice, we chose to develop a practice-based, ePRO-directed multidimensional platform to personalize as well as automate the chemotherapy-prescribing process. We hypothesized that generation of a decision support tool consisting of multiple modules focusing on the core duties preceding a chemotherapy-cycle prescription could be a more cost-effective and error-free approach. The functionalities of the tool would include chemotherapy cycle–based scheduling for data collection and analysis, an ePRO symptom collection–linked urgency algorithm, personalized symptom management, laboratory value interface, and decision support.

## Methods

### Study Design

A 1-arm, multicenter, prospective clinical trial (“Follow-up of Cancer Patients Receiving Chemotherapy or Targeted Therapy by Electronic Patient Reported Outcomes-tool” [ECHO] 7/2019-1/2021; NCT04081558) investigated the use of a novel ePRO tool, Kaiku Health, in cancer care. The most important patient inclusion criteria were colorectal cancer (CRC), a plan to receive oxaliplatin-based chemotherapy as an adjuvant therapy or in the first- or second-line setting of advanced disease, age ≥18 years, an Eastern Cooperative Oncology Group [ECOG] performance score of 0 to 2, and internet access. A decision support tool consisting of a digital symptom-monitoring tool with an urgency algorithm, laboratory values, and integration of a treatment schedule to automate chemotherapy-cycle prescribing was created for the trial.

### The ePRO Tool

The Kaiku Health ePRO follow-up module consists of a questionnaire that assess both the presence and severity of symptoms related to typical side effects of chemotherapy (blood in urine, dysuria, eye symptoms [decreased vision or other symptoms], peripheral sensory neuropathy, pain, constipation, cough, decreased appetite, diarrhea, fatigue, fever, mouth sores, nausea, rash or skin changes, shortness of breath, and vomiting). The tool also allows recording the presence of a symptom and a severity algorithm that grades the symptom according to National Cancer Institute Common Terminology Criteria for Adverse Events (NCI-CTCAE). The severity algorithm triggered an email alert to the study physician in the care unit within preset limits (the presence of a grade 3 or higher symptom or a rise in symptom severity from grade 0 to 2). The patients were informed that the ePRO follow-up was intended only for nonurgent communication, and, in critical matters, patients were advised to contact emergency care. In addition, patients received tailored, evidence-based, personalized self-care advice according to electronically reported symptoms and their grade. The tool also included a messaging option through which patients could communicate with their care team directly.

### Laboratory Values

Prior to chemotherapy infusion, peripheral blood samples were taken to evaluate bone marrow, kidney, and liver function. The laboratory values include counts for red and white cells, thrombocytes, alanine and aspartate aminotransferases, bilirubin, and creatinine. Based on the summary of product characteristics of the chemotherapeutic agents used by the patient, prespecified laboratory values were set to assess the fitness of the patient for a new course of cancer medical therapy.

### The Decision Support Tool

We developed and added a novel element to the Kaiku Health remote symptom-monitoring tool: in addition to collecting and grading ePROs, it automatically collected laboratory values via a novel interface (MyLab). The timing of the ePRO symptom questionnaires and laboratory value collection was automated according to chemotherapy cycles through another interface with a treatment-appointment reservation module (Oberon). The decision support tool analyzed the completed symptom questionnaires and preceding laboratory values and compared the results to a set of institutional reference values. The decision support tool provided a 3-tier recommendation to the care team on the initiation of the preplanned chemotherapy cycle. The 3-tier recommendations included green (meaning “go”), yellow (“evaluate and possibly postpone”), or red (“postpone”). A recommendation of go versus no-go and potential treatment visit details were sent to the patient through the Kaiku Health tool. With a no-go decision, an automated ePRO questionnaire and laboratory value collection for the rescheduled chemotherapy cycle were initiated based on the reservation module interface.

### Ethical Considerations

The study was approved by the ethics committee of the Northern Ostrobothnia Hospital District (273/2017) and registered in an international clinical study registry (ClinicalTrials.gov NCT04081558). The study was carried out in accordance with the principles of the Declaration of Helsinki and Good Clinical Practice. All patients provided written informed consent. Study participants were not compensated for their participation in the trial.

## Results

### Development of the Decision Support Tool

**Figure 1.**
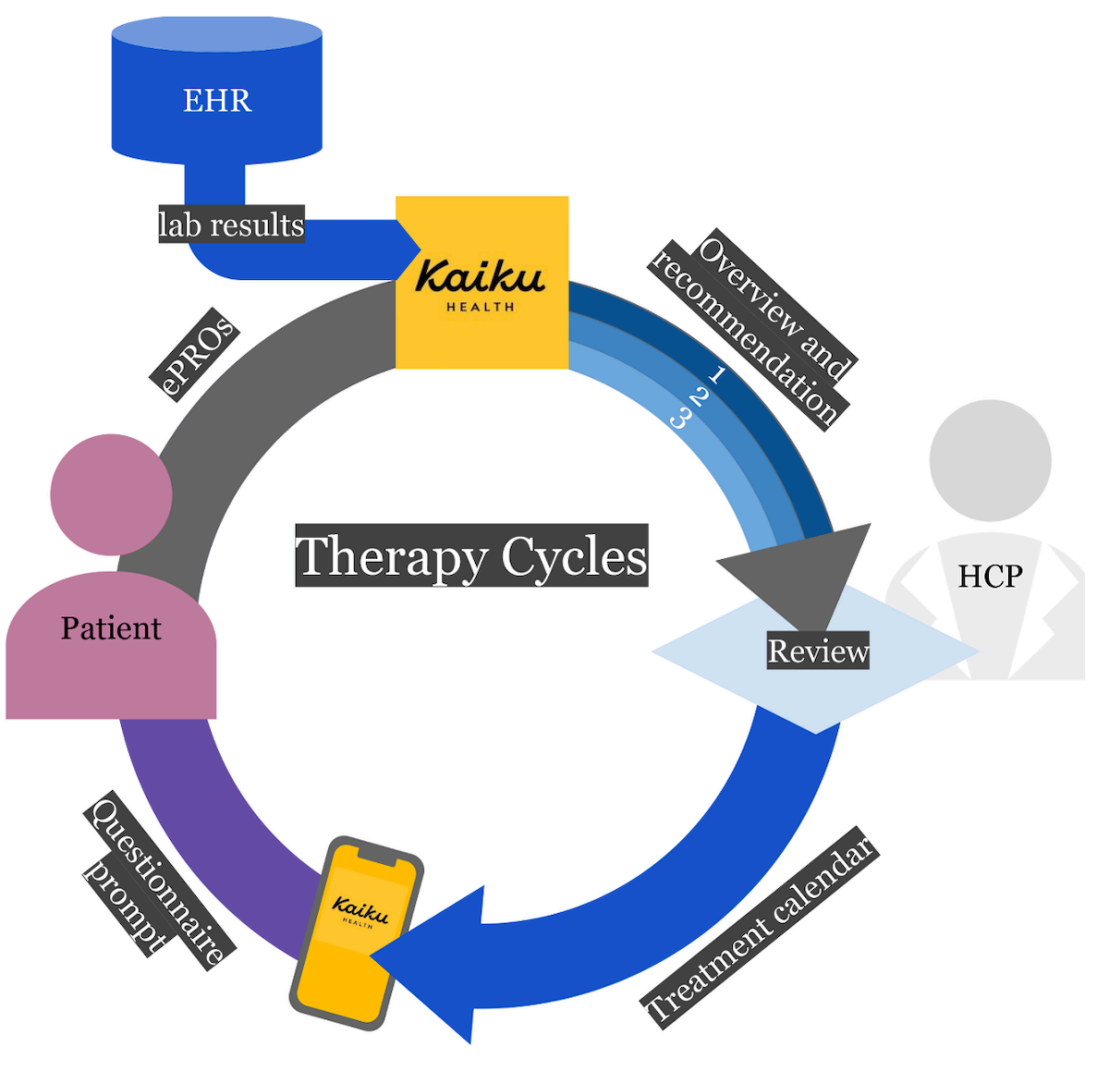
Flowchart of the methodological process of the integration of electronic patient-reported outcomes (ePROs) and laboratory values. The tool included a scheduling module that synchronized the sending of symptom questionnaires to patients via Kaiku Health and laboratory value collection from the hospital’s electronic health record (EHR) via an interface. The tool also included a symptom module with an ePRO-based questionnaire for 15 core symptoms. An evaluation module compared the symptoms and laboratory values to values in a set range and assigned a recommendation in 3 tiers (1=go; 2=hold; 3=postpone) for the chemotherapy cycle. HCP: health care professional.

Before a new chemotherapy cycle, a patient’s laboratory values and symptoms were evaluated, for example, for indications of treatable chemotherapy side effects, excess chemotherapy toxicity, and cancer progression. To streamline this work process, increase the comprehensiveness and repeatability of the precycle evaluation, and automate duties, we developed a decision support tool. The designed tool had 6 different elements that aimed to increase the efficiency and quality of cancer care (Figure 1; Multimedia Appendix 1).

**Figure 2.**
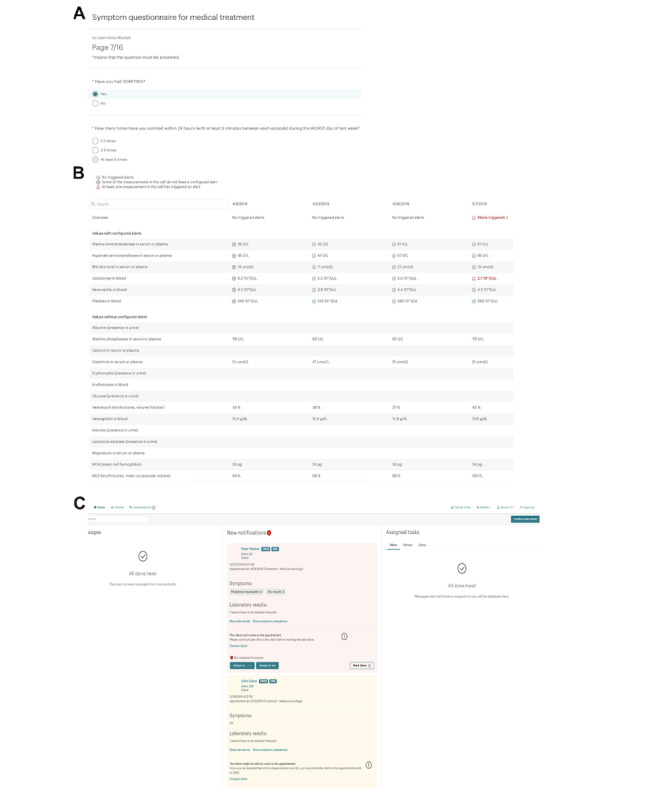
Overview of the core functionalities of the decision support tool. (A) Electronic patient-reported outcome symptom questionnaire. (B) Laboratory integration module and its output. (C) Decision support tool recommendation.

The first element was a scheduling module that synchronized the sending of symptom questionnaires to patients via Kaiku Health and laboratory value collection from hospital registries via an interface. The symptom module used ePRO-based (Kaiku Health) grading (NCI-CTCAE) for 15 core symptoms. The laboratory-value module collected specific central values via a software interface (Figure 2A). The evaluation module compared the symptoms and laboratory values to values in a set range and assigned a recommendation for the chemotherapy cycle in 1 of 3 tiers (Figure 2B). For the tier-1 (green/go) recommendation, laboratory values needed to be within a set range and all the symptoms needed to be at grade ≤1; thus, the nurse made the planned chemotherapy order to the hospital pharmacy, and an SMS text message with information on the timetable of drug infusion was sent through Kaiku Health. For the tier-2 (yellow/evaluate) recommendation, laboratory values had to be within a set range and ePROs had to include ≥1 symptom that was grade ≥2 (if they were not already at baseline); the nurse individually assessed the symptoms. If there was a need for cancer drug modification, typically dose reduction or supportive medications, the study physician was consulted as to whether the chemotherapy cycle could be initiated or modified, or if supportive medication should be initiated or modified. In the case of a “go” decision, confirmation of the treatment time was sent via Kaiku Health. In the tier-3 (red/hold) scenario, laboratory values were out of the set range, and the nurse postponed the chemotherapy cycle and sent the new infusion date through Kaiku Health. In the case of a postponed chemotherapy cycle, the scheduling module programmed a new collection of symptoms and laboratory values according to the new infusion date.

The care team could always override the recommendation of the decision support system. In the case of missing ePRO or laboratory data (or if the data were >3 days old) before the chemotherapy cycle, the decision support tool gave a tier-2 (yellow/evaluate) recommendation; thus, manual evaluation had to be done prior to prescribing the treatment. The SMS messaging module was used to inform the patient about the recommendation and potential treatment decision. Furthermore, the messaging module enabled secure messaging between the patient and care team. The symptom support module also provided the patient with personalized guidance for self-care based on the presence of specific symptoms and their grade (Figure 2C).

### Performance of Decision Support Tool–Directed Chemotherapy Scheduling

Next, we analyzed the performance of the decision support tool based on data collected in a prospective, multicenter, single-arm clinical trial (ECHO; NCT04081558). Patient recruitment for the prospective cohort took place from July 2019 to January 2021 at the Oulu University Hospital and Vaasa Central Hospital oncology units. The study dataset included patients with CRC (n=43) in the ECHO trial who gave informed consent and were treated with doublet or triplet chemotherapy in an adjuvant or metastatic setting. During the study, a total of 339 chemotherapy cycles were prescribed. Altogether, 843 ePRO symptom questionnaires were completed during the ECHO study. Of the recorded 15 symptoms, fatigue (n=446, 52.9%) and peripheral neuropathy (n=429, 50.9%) were reported most often, while 137 grade 3 or 4 symptoms were recorded, of which diarrhea (n=5, 4%) and peripheral neuropathy (n=4, 3%) were the most common. Since the functioning of the Kaiku Health symptom tool has been evaluated in multiple previous trials [16-18], we focused here on the performance of the scheduling and evaluation modules.

The function of the scheduling module was assessed by evaluating the percentage of chemotherapy cycles with sent and completed symptom questionnaires. There were no false-positive or false-negative decision support tool recommendations. For over two-thirds (n=262, 77%) of the cycles, ePRO questionnaire data were available. Preceding a new chemotherapy cycle, 65% (n=221) of cycles had symptom questionnaires graded at ≤1, while 67% (n=228) of the cycles had laboratory values in the preset range. Evaluation module recommendations were tier 1 (green/go) in 145 (42.8%) of the cycles, tier 2 (yellow/evaluate) in 83 (25%), and tier 3 (red/hold) in 111 (32.7%), while 61% of the patients (n=27) needed a phone call before any of the planned chemotherapy cycles (Table 1).

**Table 1.** Performance metrics of the developed decision support tool for automated chemotherapy-cycle prescription. The feasibility of the scheduling module was assessed by evaluating the percentage of chemotherapy cycles with sent and completed symptom questionnaires and the availability of laboratory work. The 3-tier recommendation system operated as follows: tier 1 indicated “go” (the nurse proceeded with the initial treatment plan); tier 2 indicated “hold” (the laboratory values had to be within a set range, while the reported symptoms [at least one] were grade ≥2; the study physician was consulted); and tier 3 indicated “postpone” (electronic patient-reported outcomes were acceptable, but laboratory values were outside the preset limits; the nurse scheduled the laboratory work and postponed the infusion).

Criteria	Cycles (n=339), n (%)
**Was an automated symptom questionnaire sent and completed prior to the chemotherapy cycle?**	
Yes	262 (77.3)
No	77 (23)
**Was the symptom questionnaire grading in the acceptable range prior to the planned chemotherapy cycle?**	
Yes	221 (65.2)
No	118 (34.8)
**Was the laboratory work in the acceptable range prior to the planned chemotherapy cycle?**	
Yes	228 (67.3)
No	111 (32.7)
**Evaluation module guidance (tier)**	
1	145 (42.8)
2	83 (25)
3	111 (32.7)

The experience of the health care professionals (HCPs) with the decision support system integration into the care pathway was evaluated via interviews (n=4) that explored 5 topics: improved workflow, improved care, use of resources, challenges, and points for improvement. The HCPs appreciated the improved workflow, faster patient evaluation, and direct messaging option. The laboratory module originally collected data only daily, which was considered too infrequent by the HCPs, and data collection was reprogrammed to occur multiple times a day, which improved the usability of the tool. Some inconveniences were experienced with the scheduling module for patients with Baxter pump–based treatments, for whom 2 subsequent treatment reservations were made at once (treatment and pump removal); this led to incorrect scheduling of the symptom questionnaires. The HCPs felt that the messaging option should include read notifications, which would enable verification that the patient was aware of the chemotherapy schedule.

## Discussion

### Principal Findings

We present the results of a feasibility study investigating an integrated approach with an automated decision support system for chemotherapy-cycle prescription. We show that the investigated multidimensional integrated system is feasible and can provide clinically meaningful decision support. The approach could decrease the HCP workload and increase the quality and safety of cancer care.

### Comparison to Prior Work

In recent years, the use of ePRO symptom data has gained a great deal of interest in the oncology field. Previous studies have shown that ePRO monitoring can reduce emergency room visits, improve QoL, and, more importantly, increase survival [4-6]. Integration of an ePRO system into electronic patient records is considered essential for large-scale adoption of the approach. Timely integration of data from multiple electronic health care systems into ePROs could further enhance the clinical value of the captured data and facilitate decision support.

AI-based approaches can be superior for automatic detection, classification, and interpretation using big data. However, AIs can support improper decisions if they are presented with data beyond their original training set data. One could speculate that AI-based decision support systems could surpass our approach with a simple comparison to reference values. However, chemotherapy has a narrow therapeutic window and relying on prescription decisions made by AI alone may not satisfy ethical requirements without wide-scale training and validation [19-21].

A fast track for AI integration in clinical practice could lie in the use of large language models (LLMs). Methodological developments in LLMs such as in-context learning and retrieval-augmented generation could improve LLM performance and reduce hallucinations, consequently making the use of LLMs possible in clinical practice. It has been suggested that the recent development of advanced LLMs could improve patient care across several areas, such as clinical decision support or helping to answer patients’ questions, and could even improve patient outcomes by identifying social determinants of health and reducing health disparities [22-24].

### Strengths and Limitations

In this study, we investigated a next-generation approach aiming to streamline and automate the prescription of chemotherapy cycles. We identified symptom profiles and laboratory values as central decision mediators for safe chemotherapy prescription. We hypothesized that symptom profiles could be collected via ePROs in a more comprehensive manner and used as input for a decision support tool that would also collect laboratory data and schedule collections based on the expected cycle date in an automated fashion. Furthermore, our approach included an expanded ePRO module with an integrated urgency algorithm, symptom-based personalized feedback and self-care advice, and a safe messaging platform.

We were able to show that the investigated platform is technically feasible, with good user experiences among patients and HCPs. More importantly, we found that automated “go” decisions for new chemotherapy cycles were made in almost half of the chemotherapy cycles. It is likely that this proportion could be increased by focusing only on core symptoms, instead of the total of 17 symptoms that were recorded in this study. We speculate that the investigated tool would decrease the workload for HCPs and enable allocation of human resources to more value-creating duties. We strongly believe that the computerized comparison of data to a set reference is more precise and error-free than human assessment, even though our study did not provide any metrics on the specific matter.

Nevertheless, a few modifications to the platform were suggested by end users. First, in the testing phase, the frequency of laboratory data collection was already considered too low; thus, the laboratory module was reprogrammed to search for data multiple times a day, which greatly improved the usability of the subtool. Second, the HCPs felt that the messaging option regarding the schedule of the planned chemotherapy cycle should include read notifications, which would enable verification that the patient was aware of the coming cycle. Furthermore, due to the low number of study participants and the observational nature of the study, the results are to be interpreted with caution, and the generalizability of the feasibility of the studied platform is unclear.

### Future Directions

Due to the feasibility design of the study, the results are generally hypothesis generating. Since the study investigated a complex technical tool, initial testing with a feasibility approach is therefore warranted before progressing to a larger clinical study. The integration of an LLM-based module for interactivity, such as a chat box, could enhance the supportive role of the developed tool by providing educational elements to empower patients as well as give guidance on milder-grade chemotherapy toxicity management on a more individual level. Furthermore, the rather large proportion of symptom questionnaires with grade 2 symptoms might be decreased by fine-tuning the symptom evaluation, possibly by using AI-based models to mimic diagnostic pattern recognition to identify red-flag symptoms in parallel with laboratory value assessment. To conclude, the results of our trial are promising and lay the groundwork for further studies as well as development of the platform. A randomized study design would be required to fully validate the clinical utility and cost-effectiveness of the system.

## Supplementary material

10.2196/62749 Multimedia Appendix 1 Kaiku Bridge combines the Kaiku Health symptom data from the electronic patient-reported outcome platform and laboratory value data from the hospital records (Effica) according to the scheduled care events (Oberon). After evaluating both symptoms and laboratory values within predefined limits, the Kaiku interface provides guidance in 3 tiers on new chemotherapy-cycle prescription.

## Abbreviations

AI: artificial intelligence
CDSS: clinical decision support system
CRC: colorectal cancer
ECHO: Follow-up of Cancer Patients Receiving Chemotherapy or Targeted Therapy by Electronic Patient Reported Outcomes-tool
ECOG: Eastern Cooperative Oncology Group
ePRO: electronic patient-reported outcome
HCP: health care professional
LLM: large language model
NCI-CTCAE: National Cancer Institute Common Terminology Criteria for Adverse Events
QoL: quality of life
