# Supplementary figures and images for: Development of a Comprehensive Decision Support Tool for Chemotherapy-Cycle Prescribing: Initial Usability Study
Source: JMIR Form Res. 2025 Mar 31;9:e62749. doi: 10.2196/62749 (PMC11975257; doi:10.2196/62749)

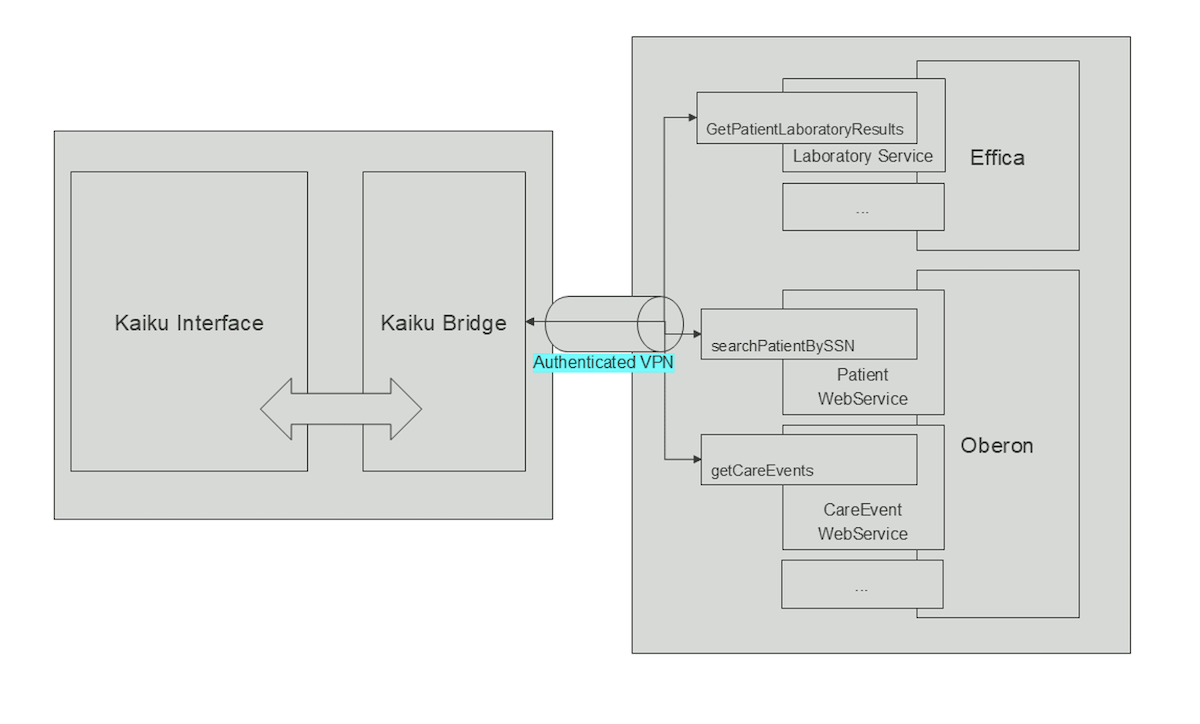

Supplement: Multimedia Appendix 1 [file formative-v9-e62749-s001.png]
